# Minimal impact of feed intolerance during therapeutic hypothermia for hypoxic ischaemic encephalopathy in a South African cohort with a standardised feeding regimen

**DOI:** 10.3389/fped.2025.1611619

**Published:** 2025-07-30

**Authors:** Ilhaam Samaai, Michael S. Pepper, Shakti Pillay, Alan R. Horn

**Affiliations:** ^1^ Department of Paediatrics and Child Health, Division of Neonatal Medicine, University of Cape Town, Cape Town, South Africa; ^2^ Department of Medical Immunology, Institute for Cellular and Molecular Medicine, Faculty of Health Sciences, University of Pretoria, Pretoria, South Africa; ^3^ Extramural Unit for Stem Cell Research and Therapy, South African Medical Research Council, Pretoria, South Africa

**Keywords:** neonate, hypoxia ischaemia -brain, hypothermia -induced, Africa south of the Sahara, neonatal encephalopathy, nutrition-enteral

## Abstract

**Introduction:**

Enteral feeding during therapeutic hypothermia (TH) for neonatal hypoxic ischaemic encephalopathy (HIE), is beneficial, but there is insufficient evidence to guide timing and feed advancement strategies. The aim of this study was to describe feed tolerance and outcomes after TH with a standardized progressive early enteral feeding regimen.

**Methods:**

Data were retrospectively reviewed from neonates with HIE who were treated with TH for HIE in the Groote Schuur Hospital (GSH) Neonatal intensive care unit (NICU), between 1 July 2019 and 31 October 2022. Enteral feeds were commenced at age 12–24 h and incremented daily if tolerated, at 12 ml/kg/day for the first 3 days and 24 ml/kg thereafter. Nutritional, morbidity and mortality outcomes were compared between neonates with and without early feed intolerance (EFI) by the fourth day of life.

**Results:**

Thirty three percent (16/48) developed EFI. However, by day six the median (IQR) enteral volumes were, 120 (110–120) and 90 (90–99), in neonates without and with feed intolerance respectively. There were no differences in resuscitation characteristics. Neonates with EFI, had higher HIE grades, more amplitude integrated electro-encephalograph (aEEG) suppression at 48 h (*p* = 0.002), later attainment of full nutritive sucking or cup feeds (*p* < 0.001) and longer hospital stays (*p* = 0.038). There were no differences in other morbidities. Mortality was 6% and necrotising enterocolitis did not occur in either group.

**Conclusions:**

Early feeding was generally well tolerated. Feed intolerance was more frequent in neonates with severe HIE, but most neonates achieved independence from IV fluids by day six.

## Introduction

Neonatal encephalopathy (NE) occurring after acute peripartum hypoxia, in the absence of other causes, is referred to as hypoxic ischaemic encephalopathy (HIE) (1). Hypoxic ischaemic encephalopathy is a major cause of neonatal mortality, multiorgan morbidity and subsequent disability, with the highest burden in low and middle-income countries (LMICS) (2). Therapeutic hypothermia (TH) is the only evidence-based therapy known to improve outcomes after moderate to severe NE due to suspected HIE (NESHIE) (3). The vascular fetal adaptive response to hypoxia—redistribution of blood to essential organs—contributes to the multiorgan morbidity which can be associated with HIE and may include necrotizing enterocolitis (NEC) (4–6). The risk for NEC in neonates with HIE is suggested by ultrasound evidence of impaired gastro-intestinal (GIT) blood flow and intestinal pathology in neonates with severe HIE (7, 8). Standard management of HIE, with or without TH, may therefore include restriction of enteral nutrition (9). Despite the potential GIT compromise associated with HIE, the most recent Cochrane systematic review showing the benefit of therapeutic hypothermia (TH) for moderate-severe HIE (10), noted that only three of the 11 included trials reported data on NEC, which occurred in only two trials; at <1% and 3.6% (11, 12). The trials which reported feeding strategies, either withheld enteral nutrition during TH (13, 14) or provided delayed and minimal enteral feeding from 24 h or later (9, 11).

However, the practice of delayed or restricted enteral feeding in term neonates with HIE, has been questioned, since it is associated with adverse outcome in preterm neonates (15). A survey of cooling centres in the United Kingdom (UK) in 2016, showed that only 41% withheld feeds during TH and 29% gave parenteral nutrition (PN) (16). Retrospective cohort studies of neonates treated with TH, comparing early minimal enteral feeding (MEF) to delayed enteral feeding (DEF), have found probable benefit of MEF including; increased breast feeding at discharge (17, 18), no difference in adverse events, NEC or feed intolerance (17–21), re­duced length of stay (18, 19), reduced time to full feeds (19, 20), and reduced proinflammatory cytokines (19). The authors of the most recent systematic review comparing the outcomes of delayed vs. early enteral feeding in infants undergoing TH for HIE, concluded that enteral feeding during TH is feasible, safe, and beneficial, but there is insufficient evidence to guide timing of initiation, size and feed advancement strategies (22).

The global knowledge gap of guidance for feeding strategies during TH is emphasized in the South African context by the variable management of early enteral nutrition for neonates receiving TH for moderate to severe HIE. The use of PN and withholding of enteral feeds was standard practice in a South African cohort described by Kali et al. (23), whereas early and progressive enteral feeding is practiced and standardized in a different South African center—the tertiary neonatal intensive care unit (NICU) at Groote Schuur Hospital (GSH) (24). The aim of this study was to provide novel South African data describing feed tolerance, characteristics and outcomes in a cohort of neonates with HIE who were cooled and managed with a standardised progressive early enteral feeding protocol at GSH. The primary objective was to describe the incidence of early feed intolerance (EFI) and the characteristics of affected neonates, during TH for moderate-severe NESHIE. The secondary objectives were: (i) To determine if there are associations between EFI and maternal or neonatal characteristics at birth; (ii) To determine if there are associations between EFI and major morbidities; and (iii) To determine if there are associations between EFI and short-term outcomes.

## Methods

### Study setting, design and ethical approval

This retrospective, descriptive, study was conducted on data from the GSH Neonatal Unit, a 75-bedded tertiary referral centre for the Metro West Area of Cape Town, with approximately 2000 admissions per annum. The study was approved by the Human Research Ethics Committee (HREC) of the Faculty of Health Sciences (FHS), University of Cape Town (UCT), (HREC 658/2024) and by the GSH hospital management. The study was developed and carried out in accordance with the Declaration of Helsinki, 2013. The study population was a cohort of neonates with HIE who were admitted to GSH, treated with TH, had data collected into the GSH neonatal encephalopathy registry (HREC 087/2010) and were also included, with their consent, in a separate observational national study; Genetic predisposition to death and disability after moderate-severe Neonatal Encephalopathy due to suspected HIE (NESHIE) in cooled infants (HREC 622/2018)—The National NESHIE Study. Data were included from neonates admitted from 1 July 2019 to 31 October 2022, representing the period with the most complete data entry for GSH neonates at the time of study design.

Retrospective collection of de-identified patient data into the GSH neonatal encephalopathy registry, of all neonates with HIE who were admitted to GSH and treated with TH, was approved by the HREC FHS UCT (HREC 087/2010). Informed consent was not required for retrospective analysis of this de-identified data, however consent was collected for all patients, for the collection of observational data as part of the procedures for the The National NESHIE TH Study.

**Inclusion Criteria for the National NESHIE TH Study, and this study.**

All of the following inclusion criteria (a), (b), (c), and (d) were required:

(a) Gestational age ≥36 weeks at birth
(b) Birth weight ≥1,800 g
(c) Intrapartum hypoxia suggested by:
  (i) Cord blood or neonatal blood in first hour of life with pH ≤ 7 or base deficit (BD) ≥ 16 mmol/L, *or*
  (ii) A history of sentinel event and first hour pH ≤ 7.15 or BD ≥ 10 mmol/L, *or*
  (iii) Intermittent positive pressure ventilation (IPPV) at 10 min
(d) Moderate or severe encephalopathy by age 6 hours based on:
  (i) moderate modified Sarnat grade or a Thompson HIE Score ≥7 (25–28), *and*
  (ii) Abnormal amplitude-integrated electro encephalograph (aEEG) (29, 30).

**Exclusion Criteria for the National NESHIE TH Study, and this study.**

Neonates with any of the following criteria were excluded:

• Consent for National NESHIE study not available
• Not treated with TH within 6 h from birth
• Moribund or too unstable to cool because of severe or unresponsive persistent pulmonary hypertension (PPHN)/hypoxia/hypotension/bleeding or hypoglycemia.
• Suspected or confirmed congenital infections
• Chromosomal abnormalities or major congenital anomalies affecting neurological outcome

In addition to the above criteria, neonates with missing key data describing characteristics, outcomes and/or feed and fluid management, were excluded from this study.

### Standard medical care and feeding definitions

All included neonates were cooled to a core rectal target temperature of 33.5°C for 72 h, using an automated Blanketrol III (Cincinnati Sub-Zero, Cincinnati, Ohio) cooling mat. During rewarming the rectal temperature was increased by 0.2°C per hour until a temperature of 36.5°C was reached. Neonates were sedated with morphine during cooling unless already obtunded, comatose or sedated with anticonvulsants. Heart rate and oxygen saturations were monitored continuously, and blood pressure was documented hourly. Investigation on admission included blood glucose, electrolytes haemoglobin (Hb) and blood culture. First-line antibiotics were commenced on admission but stopped at 36–48 h if infection indicators were negative. A cranial ultrasound scan was performed to exclude other causes of NE.

Intravenous (IV) fluids were initiated at 40 ml/kg/day, with a potassium-free solution containing 10% dextrose, 33 mmol/L sodium chloride and 5 mmol/L calcium gluconate [Sabax Potassium-free Neonatal maintenance Solution, (Adcock Ingram, Midrand, South Africa)]. Total administered fluids (IV and enteral combined) were maintained at 40 ml/kg/day for the first three days—or adjusted according to sodium levels—and increased progressively thereafter by 20–30 ml/kg/day to a final total of 150 ml/kg/day. If hypoglycaemia occurred, the concentration of administered dextrose was increased rather than volume. Enteral feeds were commenced at age 12–24 h, using term formula milk or preferably expressed breast milk (EBM) if available, since EBM was frequently not available, particularly when neonates were outborn. Feeds were incremented daily if tolerated at 12 ml/kg/day for the first 3 days and 24 ml/kg thereafter.

Feed intolerance was defined by the presence of one or more of the following: tense abdominal distention, recurrent vomiting, and/or bloody stools. Early feed intolerance was defined as, a tolerated enteral feed volume on day four, which was less than the volume which would be expected to be attained by day three (<36 ml/kg/day), due to any cause of feed intolerance as defined above.

### Encephalopathy assessment methods and aEEG

The clinical grade of HIE was assessed before TH and daily with both the Thompson HIE score (27), and the Modified Sarnat Grade (25, 26). The Thompson HIE score consists of nine clinical signs, scored numerically to a maximum of 22, and a score a of ≥7 was considered to indicate moderate-severe HIE (27). When HIE was assessed by the Modified Sarnat Grade, moderate-severe HIE was defined clinically by the presence of one or more abnormal signs in at least three of six possible clinical categories or the presence of seizures, where the grade severity is determined by the number of signs per grade, and if these are equal, the grade is determined by the level of consciousness (25, 26). An abnormal aEEG was required in addition to clinical signs, to assign a diagnosis of moderate-severe HIE by age 6 h. The aEEG background was classified according to pattern, into continuous normal voltage (CNV), discontinuous normal voltage (DNV), burst suppression (BS), continuous low voltage (CLV), and flat trace (FT)—aEEGs with abnormal backgrounds (DNV, BS, CLV, or FT) and/or those showing seizures were considered abnormal (29, 30). A suppressed background was defined by backgrounds showing BS, CLV or FT which aligns with voltage criteria for suppression described by al Naqeeb et al. (31) and is strongly associated with abnormal neurodevelopmental outcome or death, when present at age 48 h in cooled neonates with moderate-severe HIE (32).

### Sample size and data collection

The sample was defined by the number of neonates admitted to GSH with moderate to severe HIE and treated with TH, who were also included in the separate National NESHIE study, at the time of study design, which spanned a period of 39 months. De-identified data were routinely and prospectively collected on all cooled neonates admitted to GSH and entered into an access-controlled Research Electronic Data Capture (REDCap) database, accessible to the departmental research clinicians. Identifying data is kept in a separate password-protected Excel spread sheet, accessible only to senior clinician database managers who ordinarily manage the cooled neonates (limited to the senior authors, ARH and SP). The GSH registry also includes the deidentified study numbers for patients who are included in related trials, including The NESHIE Study. The following de-identified data of patients who were included in the NESHIE Study, were extracted from the ethics-approved REDCap registry (HREC 087/2010) directly into a statistical database using STATA 18.0 BE-Basic edition (StataCorp, Texas, USA):

(i) Baseline maternal, antenatal, labour and neonatal characteristics at birth and before cooling; (ii) Neonatal neurological assessments before cooling, at ages 24 and 48 h and maximum scores during admission; (iii) Fluid and nutrition management during the first four days of life; (iv) Major morbidity data including the occurrence of hypoglycaemia (<2.6 mmol/L), hyperglycaemia (>8.3 mmol/L), hyponatraemia (<130 mmol/L), serum creatinine at 24 h, anaemia (Hb ≤ 12 g), bleeding treated with blood products, mechanical ventilation, FiO_2_ on admission, hypotension requiring inotropes, early sepsis (positive blood or cerebrospinal fluid culture ≤72 h after birth), late sepsis (positive blood or cerebrospinal fluid culture >72 h after birth), and NEC (modified Bell's stage II or III NEC, shown by abdominal distension, gastric aspirate *and/or* blood in stools together with abdominal x-ray showing bowel oedema, pneumatosis or pneumoperitoneum) (33); and v) Short-term outcome data including EFI (a tolerated enteral feed volume on day four, which was less than the volume which would be expected to be attained by day three; <36 ml/kg/day), durations of hospital stays, ages at suck/cup feeding, maximum Thompson scores, maximum modified Sarnat grades, suppressed aEEG at 48 h, and mortality.

### Data analysis

Differences in characteristics, morbidity and short-term outcomes, between neonates with and without early feed tolerance were analysed using STATA 18.0 BE-Basic edition (StataCorp, Texas, USA). Categorical variables were analysed with Chi square or Fischer's exact tests and continuous variables were analysed with student t-test or Wilcoxon rank sum, with significance assigned as *p* < 0.05.

## Results

**Figure 1:**
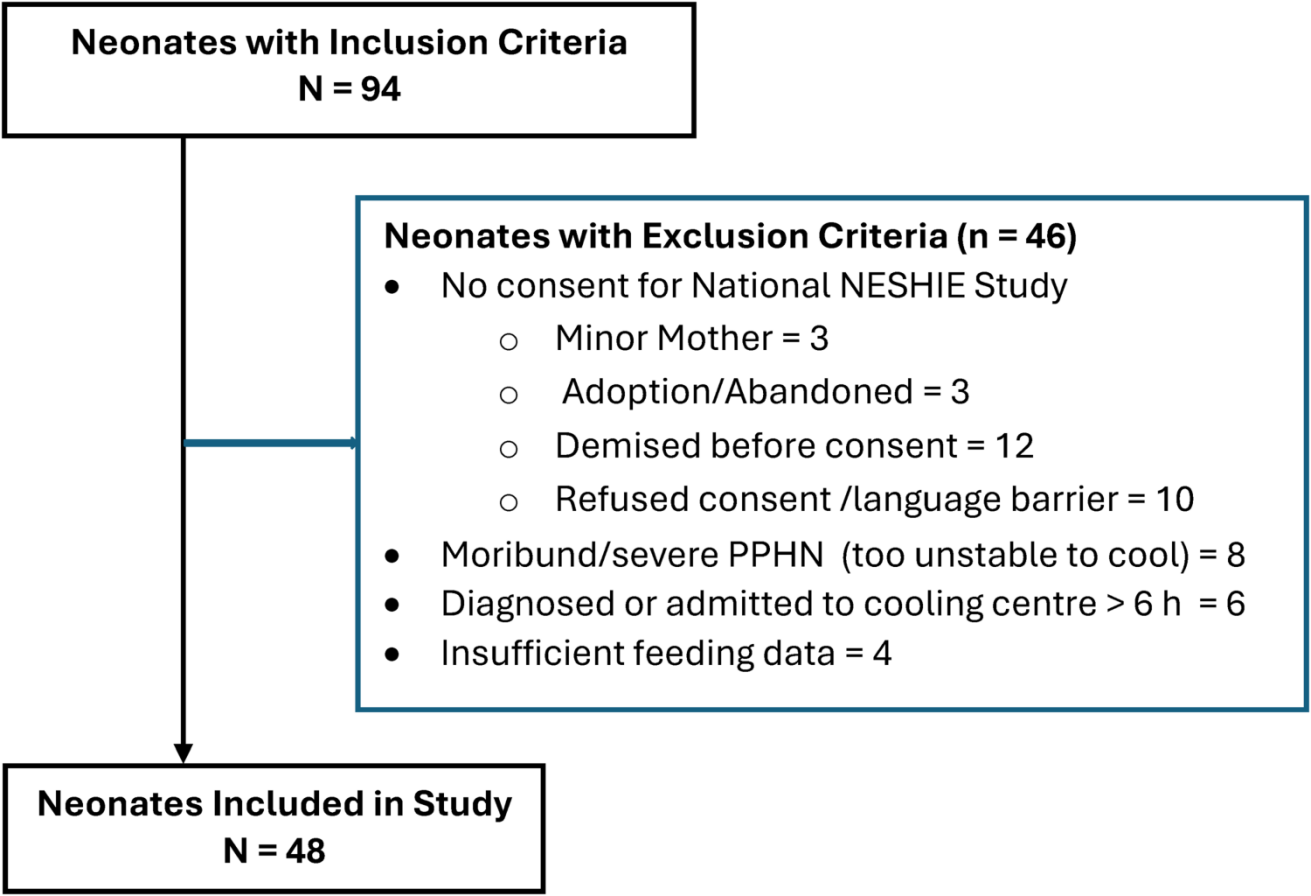
Application of inclusion and exclusion criteria. H, hours; PPHN, persistent pulmonary hypertension.

The application of inclusion and exclusion criteria is shown a flow chart in Figure 1. Forty-eight neonates with complete data were included in our cohort, after 46 neonates were excluded. The perinatal characteristics, grouped according to EFI, are shown in Table 1. Sixteen (33%) neonates in the study had EFI. There were no differences in perinatal characteristics. Notably, sentinel events were only reported in 34%, a minority of neonates were inborn (15/48; 31%), the mean ± SD weight was 3,035 ± 420 g, the median (IQR) 5 min Apgar was 5 (4–6) and the median (IQR) base deficit (BD) was 15.2 (19.2–10.5).

**Table 1:** Perinatal characteristics grouped according to feed intolerance.

Characteristics	Total *N* = 48 *Median (IQR),* *n (%)* or *mean (SD)*	No EFI *N* = 32 *Median (IQR),* *n (%)* or *mean (SD)*	EFI *N* = 16 *Median (IQR),* *n (%)* or *mean (SD)*	*P* value
Maternal characteristics				
Age (years)	25 (20.5–30)	23 (19.5–29.5)	28 (25–31)	0.079
Gravidity	1 (1–2)	1 (1–2)	2 (1–3)	0.072
Illicit/alcohol/smoked	5 (10%)	4 (13%)	1 (6%)	0.652
Antenatal morbidity present	24 (50%)	17 (53%)	7 (43%)	0.540
Chorioamnionitis	0			
Sentinel events	16 (34%)	10 (32%)	6 (38%)	0.719
Prolonged 2nd Stage >2 h	7 (15%)	5 (16%)	2 (13%)	1.000
Vaginal delivery	31 (65%)	21 (65%)	10 (63%)	1.000
Neonatal characteristics at birth				
Inborn	15 (31%)	11 (34%)	4 (25%)	0.742
Male	35 (73%)	25 (78%)	10 (63%)	0.310
Weight (grams)	3,035 (420)	3,035 (412)	3,033 (448)	0.990
Apgar at 1minute	2 (1–4)	2 (1–4)	2 (1–3)	0.346
Apgar at 1minute	5 (4–6)	5 (4–6)	5 (4–5)	0.293
Chest Compressions given	14 (29%)	10 (31%)	4 (25%)	0.746
pH *(n* *=* *40;27;13)*^a^	7.03 (0.18)	7.04 (0.19)	7.01 (0.16)	0.690
Base Deficit (mmol/L) *(n* *=* *40)*^a^	15.2 (19.2–10.5)	15.3 (19.2–9.4)	15.0 (19.1–12.1)	0.619

EFI, early feed intolerance; IQR, interquartile range; IV, intravenous; SD, standard deviation.

^a^ Early blood gas available for 40 neonates: 27 without EFI and 13 with EFI.

The fluid and nutritional management data, grouped according to EFI, from day 1–4 and enteral feeds on day 7, are shown in Table 2. Total fluid intake did not differ between groups on day 1–3, however on day 4 those with EFI received 20 ml/kg more fluid (*p* = 0.003). There were no differences in enteral feeds on day 1 and 2 between both groups, however significantly less feeds were given (tolerated) on days 3 and 4 in the EFI group (*p* < 0.001)—this difference persisted to day 7, where those with EFI were only receiving 120 ml/kg/d compared to 150 ml/kg/d in those without EFI (*p* = 0.003). Only one baby required PN.

**Table 2:** Fluids and nutrition grouped according to feed intolerance.

Fluids and nutrition	Total *N* = 48 *Median (IQR),* *n (%),* or *mean (SD)*	No EFI *N* = 32 *Median (IQR),* *n (%,)* or *mean (SD)*	EFI *N* = 16 *Median (IQR),* *n (%,)* or *mean (SD)*	*P* value
Total fluids				
Day 1	40 (40–40)	40 (40–40)	40 (40–40)	1.000
Day 2	40 (40–40)	40 (40–40)	40 (40–40)	1.000
Day 3	40 (40–40)	40 (40–40)	40 (40–40)	1.000
Day 4	52 (40–60)	40 (40–51)	60 (43–70)	0.003
Enteral milk ml/kg/day				
Day 1	0	0	0	
Day 2	12 (11–20)	12 (12–21)	12 (10–17)	0.380
Day 3	24 (20–36)	27 (22–40)	20 (12–24)	< 0.001
Day 4	40 (32–60)	57 (40–60)	29 (21–32)	< 0.001
Day 5 *(n* *=* *46;31;15)*^a^	80 (60–90)	90 (76–90)	60 (48–65)	< 0.001
IV fluids Day 5	8/46 (17.4%)	2/31 (6.5%)	6/15 (40%)	0.01
Day 6 *(n* *=* *44;30;14)*^b^	118 (90–120)	120 (110–120)	90 (90–99)	0.002
IV fluids Day 6	4 (9.1%)	1/30 (3.3%)	3/14 (21.4%)	0.088
Day 7 *(n* *=* *44;30;14)*^b^	149 (120–150)	150 (140–150)	120 (120–128)	0.003
IV fluids Day 7	2 (4.6%)	1 (7.1%)	1 (3.3%)	0.54
Parenteral Nutrition				
Day 1 to Day 4	1 (2%)	0	1 (6%)	0.333

EFI, early feed intolerance; IQR, interquartile range; IV, intravenous; SD, standard deviation.

^a^ Two neonates died before assessment.

^b^ Two neonates died and two neonates transferred before assessment.

**Figure 2:**
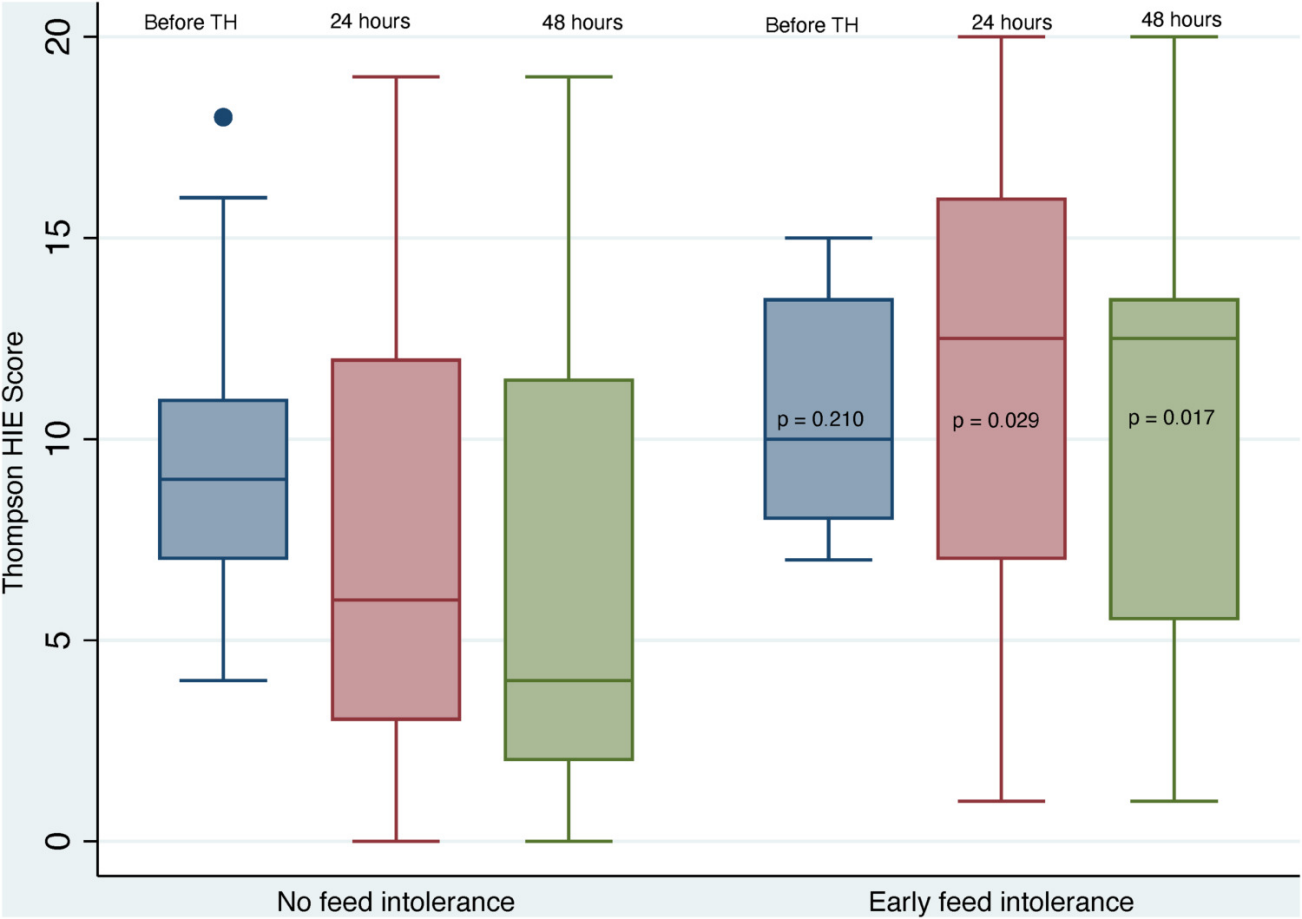
Early thompson scores grouped according to feed tolerance. HIE, hypoxic ischaemic encephalopathy; TH, therapeutic hypothermia.

The differences in neurological assessments, grouped according to EFI, are shown in Table 3. There were no differences in pre-cooling Thompson scores between groups, however considerably higher Thompson scores were found in the EFI group at 24 and 48 h (*p* = 0.029 and 0.017 respectively) and these neonates also had significantly higher maximum Thompson scores (*p* = 0.003)—these data are depicted graphically in Figure 2. Assessment with the Modified Sarnat grade, showed higher HIE grades in the neonates with EFI at all stages of assessment, and the maximum HIE grade was most often grade 3 [median (IQR): 3 (3–3), *p* = 0.002].

**Table 3:** Neurological assessments grouped according to feed tolerance.

Neurological assessment	Total *N* = 48 *Median (IQR), n (%)* or *mean (SD)*	No EFI *N* = 32 *Median (IQR), n (%)* or *mean (SD)*	EFI *N* = 16 *Median (IQR), n (%)* or *mean (SD)*	*P* value
Thompson HIE Score				
Before TH	9 (7–12)	9 (7–11)	10 (8–13)	0.210
24 h	7 (4–13)	6 (3–12)	12 (7–16)	0.029
48 h	5 (2–13)	4 (2–11)	12 (5–13)	0.017
Maximum	12 (8–16)	9 (7–13)	14 (13–16)	0.003
Modified sarnat grade				
Before TH	2 (2–2)	2 (2–2)	2 (2–3)	0.009
24 h	2 (1–3)	2 (1–3)	3 (2–3)	0.020
48 h	2 (1–3)	1 (1–2)	3 (2–3)	0.005
Maximum	2 (2–3)	2 (2–3)	3 (3–3)	0.002
aEEG background *(0* *=* *FT; 1* *=* *BS; 2* *=* *CLV; 3* *=* *DNV; 4* *=* *CNV)*				
Before TH	3 (3–4)	4 (3–4)	3 (2–3)	0.051
24 h	3 (2–4)	3 (2–4)	2 (0–3)	0.020
48 h	3 (2–4)	3 (3–4)	2 (0–3)	0.015
Suppressed aEEG at 48 h	16 (33%)	6 (19%)	10 (63%)	0.002
Seizures				
Day 1	28 (58%)	15 (47%)	13 (81%)	0.031
Day 2	25 (52%)	14 (44%)	11 (69%)	0.132
Day 3	10 (21%)	5 (16%)	5 (31%)	0.267

aEEG, amplitude integrated electro encephalograph; BS, burst suppression; CLV, continuous low voltage; CNV, continuous normal voltage; DNV, discontinuous normal voltage; EFI, early feed intolerance; FT, flat trace; HIE, hypoxic ischaemic encephalopathy; IQR, interquartile range; IV, intravenous; SD, standard deviation; TH, therapeutic hypothermia.

A numeric value was aligned with the aEEG background, where “0” is most severe and “4” is normal. The neonates with EFI had more severe aEEG background patterns at 24 and 48 h and at 48 hours they had the highest proportion of suppressed aEEG background (63% vs. 19% in those without early feed intolerance; *p* = 0.002). The neonates with EFI had more seizures prior to cooling (81% vs. 47%; *p* = 0.031), but the difference did not persist.

The morbidity and short-term outcomes, grouped according to EFI, are shown in Table 4. There were no differences in the proportions of morbidities between groups—notably, 35% required mechanical ventilation and 21% required inotropes, but early and late sepsis occurred in only 2% and 4% respectively and NEC did not occur. However, the EFI group had longer hospital stays (*p* = 0.038), higher Thompson scores (*p* = 0.003), higher Sarnat grades (*p* = 0.002) and 3 times more frequent suppressed aEEG at 48 h (*p* = 0.002). Only 6% of neonates died, and there were no differences in mortality between the groups.

**Table 4:** Morbidity and short-term outcome grouped according to feed tolerance.

Morbidity and outcome	Total *N* = 48 *Median (IQR),* *n (%)* or *mean (SD)*	No EFI *N* = 32 *Median (IQR),* *n (%)* or *mean (SD)*	EFI *N* = 16 *Median (IQR),* *n (%)* or *mean (SD)*	*P* value
Morbidity				
Hypoglycaemia ever	14 (29%)	8 (25%)	6 (38%)	0.503
Hypoglycaemia before cooling	8 (17%)	5 (16%)	3 (19%)	1.000
Hyperglycaemia before cooling	10 (21%)	6 (19%)	4 (25%)	0.716
Hyponatraemia	14 (29%)	8 (25%)	6 (38%)	0.503
Creatinine at 24 hours *(n* *=* *39; 24; 15)*^a^	88 (66–102)	87 (71–96)	99 (57–115)	0.613
Hb < 12 g/dl ever	3 (6%)	2 (6%)	1 (6%)	1.000
Bleeding + Blood products	2 (4%)	0	2 (13%)	0.106
Mechanical ventilation	17 (35%)	11 (34%)	6 (38%)	1.000
FiO_2_% on admission	21 (21–35)	21 (21–30)	21 (21–28)	0.433
Hypotension and inotropes	10 (21%)	6 (19%)	4 (27%)	0.704
Early sepsis	1 (2%)	0	1 (6%)	0.333
Late sepsis	2 (4%)	1 (3%)	1 (6%)	0.541
Necrotising enterocolitis	0			
Outcome				
Hospital-stay in days *(n* *=* *44;30;14)*^b^	14 (10–21)	12 (9–18)	18 (13–24)	0.038
Age at nutritive suck/cup (*n* *=* *41; 27;14)*^c^	8 (5–13)	6 (5–11)	13 (8–16)	< 0.001
Maximum Thompson score	12 (8–16)	9 (7–13)	14 (13–16)	0.003
Maximum sarnat grade	2 (2–3)	2 (2–3)	3 (3–3)	0.002
aEEG suppressed at 48 h	16 (33%)	6 (19%)	10 (63%)	0.002
Death	3 (6%)	1 (3%)	2 (13%)	0.254

aEEG, amplitude integrated electro encephalograph; EFI, early feed intolerance; FiO_2_, fraction of inspired oxygen; HIE, hypoxic ischaemic encephalopathy; IQR, interquartile range; IV, intravenous; SD, standard deviation; TH, therapeutic hypothermia.

^a^ Creatinine at 24 h available for 39 neonates: 24 without EFI and 15 with EFI.

^b^ Two neonates died and two neonates transferred before assessment.

^c^ Two neonates died and missing data for five others.

## Discussion

This observational study of 48 neonates who were treated with TH for moderate-severe HIE, and were progressively enterally fed during TH, showed that EFI by day 4 of life, occurred in a third of neonates, and was strongly associated with the severity of encephalopathy at admission and 48 h, but was not associated with perinatal characteristics at birth nor with major morbidities. Most neonates did not require IV fluids after day 5, irrespective of early feed intolerance and NEC did not occur.

### Necrotising enterocolitis

The low rate of NEC with early enteral feeding during TH is well described, and previous studies described NEC rates varying from 0%–4.3% when defined as Bell stage II or higher—the only study reporting NEC rate above 0%, included neonates at 35 weeks’ gestation, however two other studies including neonates at ≥35 weeks’ gestation, reported 0% NEC (19–21, 34, 35). The inclusion of neonates at 35 weeks’ gestation in some studies, may limit comparison, since feeding intolerance is more frequent in preterm neonates (15). However the mean gestational age in studies which included neonates at 35 weeks’ gestation, was ≥38 weeks’ gestation (19, 20, 35). A large population-level retrospective cohort study of 6,030 neonates with HIE and TH, from the UK National Neonatal Research Database found that severe NEC, confirmed at surgery or causing death occurred in 0.1% (18). The low rate of NEC in this context is in keeping with data from very low birthweight infants managed with early MEF, which was associated with increased GI motility, increased GI hormone secretion, decreased GI inflammation, more beneficial microflora, improved GI tract maturation and decreased feeding intolerance without increasing the risk of developing NEC (15). Moreover, neonates with moderate-to-severe HIE who are treated with TH have less feeding intolerance compared to non-cooled neonates (17, 19, 36).

### Feeding intolerance and time to full enteral feeding

Few studies of feeding and TH have reported the rates of feeding intolerance. Similar to the 33% EFI in our study, Hu et al. (35), reported 26% feeding intolerance, in the early enteral feeding group in their RCT, where enteral feeds were commenced and advanced daily with “<20 ml/kg/day” breast milk/formula, at a mean ± SD age of 2 ± 0.91 days; compared to 32% feeding intolerance in the delayed group, where enteral feeds were commenced 4.84 ± 0.97 days. Severe HIE occurred in 33% of neonates in both studies, and although Hu et al. included neonates at 35 weeks’ gestation, the mean gestation in the early feeding group was 39.9 weeks. Feeding intolerance was reported in only 9% of neonates in a retrospective multicentre five-year review of minimal enteral nutrition (MEN) during TH (21). However, only 12% had severe HIE, and the feeding strategy during TH was not described.

Time to full enteral feeding (TTFEF) can be considered as an indirect indication of feed tolerance. Several studies have defined and reported TTFEF with early enteral feeding during TH, but with substantial variation in the definition of TTFEF; 150 ml/kg/day for at least three consecutive days (mean ± SD TTFEF; 7.6 ± 3.5 days) (20), “no requirement for intravenous fluid” [median (IQR) TTFEF; 7 (5–11) days] (34), 120 ml/kg/day (mean ± SD TTFEF; 9.91 ± 1.88 days) (35), 120 ml/kg/day for 24 h [median (IQR) TTFEF; 6 (5–9) days] (21), and “time between birth and the time when parenteral nutrition was terminated” [median (IQR) TTFEF 6 (3–19) days] (17). The early enteral feeding volumes and strategies in these studies were variable; feed with 12–24 ml/kg/day if not hypotensive, coaguloapthic, hypoxic or acidotic—usually on or after the third day of life (20), initiate and advance feeds at 10–20 ml/kg/day after 24 h as tolerated when considered “stable” (34), initiate and advance feeds as tolerated at <20 ml/kg/day*,* if “medically feasible” during TH/rewarming (35), start “MEN after 12–24 hours of life” (21), and feeds initiated at median 23.6 h with 1–2 ml/kg/bolus every 3 h (8–16 ml/kg/d) and increased if tolerated (17).

In our study, a regimen of 3-hourly bolus feeds at 12 ml/kg/day, initiated after 12–24 h, resulted in median enteral feed volumes of 120 ml/kg/day by day 6 in neonates without EFI, and by day 7 in neonates with EFI. By day 6 of life, only 3% and 21%, of neonates without and with EFI respectively, were receiving IV fluids. These data suggest that our cohort, including the neonates with feed intolerance, had comparable or faster TTFEF, when compared to other studies. The tolerance of feeds and low proportion of neonates receiving IV fluids by day 6 in our cohort, may be related to our protocol of restricted total fluid volume of 40 ml/kg/day for the first three days, which is different to some centres which administer 60 ml/kg/day (37). While more evidence is needed to guide the use of fluid restriction, our approach avoids a positive fluid balance which has been associated with death and increased severity of brain injury in neonates with HIE (38, 39).

### Feeding intolerance and disease severity

Previous studies have reported an increased frequency of delayed feeding initiation and/or delayed attainment of full enteral feeding in neonates with severe HIE and/or cardio-respiratory instability, including mechanical ventilation and inotrope use (20, 34, 37, 40). We found a similar association with severe HIE. The neonates with EFI in our study had significantly higher Thompson HIE scores and Modified Sarnat HIE Grades than neonates without EFI, and they had a three times higher frequency of suppressed aEEG at 48 h. The predominance of severe HIE in neonates with EFI in our study, also aligned with their prolonged hospital stay and later age of attaining nutritive suck/cup feeding. The progressively increasing difference between Thompson HIE scores of neonates with and without EFI, before and during cooling (Figure 2), may represent disease progression and less response to TH in the neonates with severe HIE and EFI.

In addition to the anti-inflammatory effects of TH, it may reduce GI injury after hypoxia-ischaemia, by modulating or reducing the excessive GI blood flow which can occur during the reperfusion phase of injury in neonates with moderate HIE—however, severe HIE is associated with decreased GI blood flow which may not be improved by TH (7, 8). This mechanism may be related to the increased EFI in neonates with severe HIE. There is ongoing research into the potential value of doppler studies of GIT blood flow velocities, to assess intestinal health and guide enteral feeding approaches (41, 42).

We did not find an association between mechanical ventilation, inotrope use, or other morbidities and EFI—this may be related to our standardised approach of initiating enteral feeds in all neonates with a normal abdominal examination, irrespective of ventilation or inotrope requirement. The variable morbidity and EFI in other TH and feeding studies may be affected by the severity of intrapartum hypoxia reflected in the variable requirement of early acidosis as an inclusion criteria for cooling, where BD can vary from ≥10 to ≥16; comparisons will be more meaningful if global and/or regional criteria for TH are standardised (28). We found no association between maternal characteristics and neonatal characteristics at birth, including Apgar scores and blood gas analysis—suggesting that the extent of individual foetal/neonatal response or (mal)adaptation to the hypoxic-ischaemic exposure is more predictive than the exposure alone.

### Limitations and strengths

The primary limitation of this study is that it is a retrospective analysis of data where the sample size was determined by time and completeness relative to a separate study—it is therefore subject to selection bias and may lack power for variables with high or low frequencies. A second major limitation is the lack of data on the type of milk per feed—breast milk vs. formula—which may influence feed tolerance. However, the data represent the real-world scenario of limited availability of EBM, the data were collected prospectively, and the study provides novel descriptive data from a setting with a standardized and well-established protocol for nutritional and fluid management of HIE.

## Conclusion

Our study addresses a knowledge gap identified by studies showing benefit of early enteral nutrition in neonates with HIE who are treated with TH including a systematic review (22) and several subsequent studies (20, 21, 37)—to provide data and outcomes for specific feeding strategies. It is the first study from South Africa to provide nutritional outcome data in the context of standardised early and progressive feeding of neonates with HIE who are treated with TH.

The progressive, standardised, early feeding regimen of neonates at GSH with HIE who were treated with TH, was generally well tolerated. Independence from IV fluids was achieved by neonates in this study, at comparable or earlier times than were observed in other studies. Early feed intolerance was more frequent in neonates with severe HIE, but most neonates did not require IV fluids after day 5 of life, NEC did not occur and there was a low mortality rate. Further studies should focus on accurately quantifying and standardizing the approach to enteral feeds for neonates, including the use of EBM and donor EBM, according to severity of HIE.

## Data Availability

The datasets presented in this article are not readily available because informed consent was not taken for that type of data storage and access. However, access to the de-identified raw data set, for purposes of collaborative sub-studies with the authors, pending relevant ethics committee approval, can be obtained from the corresponding author. Requests to access the datasets should be directed to alan.horn@uct.ac.za.
